# Ethyl 5-formyl-2,4-dimethyl-1*H*-pyrrole-3-carboxyl­ate

**DOI:** 10.1107/S1600536808014542

**Published:** 2008-05-21

**Authors:** Si-Shun Kang, Hai-Lin Li, Hai-Su Zeng, Hai-Bo Wang

**Affiliations:** a College of Science, Nanjing University of Technolgy, Xinmofan Road No.5, Nanjing 210009, People’s Republic of China

## Abstract

The molecule of the  title compound, C_10_H_13_NO_3_, is approximately planar. A network of N—H⋯O and weak C—H⋯O hydrogen bonds helps to consolidate the crystal structure.

## Related literature

For related literature, see: Sun *et al.* (2002 ▶). For details of the synthesis, see: Tang *et al.* (1999 ▶).
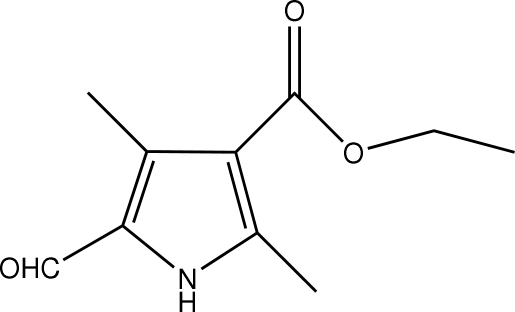


## Experimental

### 

#### Crystal data

C_10_H_13_NO_3_
*M* *_r_* = 195.21
Monoclinic, 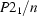

*a* = 3.9830 (8) Å
*b* = 15.572 (3) Å
*c* = 16.213 (3) Å
β = 96.96 (3)°
*V* = 998.2 (3) Å^3^
*Z* = 4
Mo *K*α radiation
μ = 0.10 mm^−1^
*T* = 293 (2) K
0.20 × 0.05 × 0.05 mm

#### Data collection

Enraf–Nonius CAD-4 diffractometer
Absorption correction: ψ scan (North *et al.*, 1968 ▶) *T*_min_ = 0.981, *T*_max_ = 0.995
2069 measured reflections
1798 independent reflections
935 reflections with *I* > 2σ(*I*)
*R*_int_ = 0.021
3 standard reflections every 200 reflections intensity decay: none

#### Refinement

*R*[*F* ^2^ > 2σ(*F* ^2^)] = 0.083
*wR*(*F* ^2^) = 0.190
*S* = 1.03
1798 reflections
127 parameters
H-atom parameters constrained
Δρ_max_ = 0.16 e Å^−3^
Δρ_min_ = −0.17 e Å^−3^

### 

Data collection: *CAD-4 Software* (Enraf–Nonius, 1989 ▶); cell refinement: *CAD-4 Software*; data reduction: *XCAD4* (Harms & Wocadlo, 1995 ▶); program(s) used to solve structure: *SHELXS97* (Sheldrick, 2008 ▶); program(s) used to refine structure: *SHELXL97* (Sheldrick, 2008 ▶); molecular graphics: *SHELXTL* (Sheldrick, 2008 ▶); software used to prepare material for publication: *SHELXL97*.

## Supplementary Material

Crystal structure: contains datablocks global, I. DOI: 10.1107/S1600536808014542/hb2732sup1.cif

Structure factors: contains datablocks I. DOI: 10.1107/S1600536808014542/hb2732Isup2.hkl

Additional supplementary materials:  crystallographic information; 3D view; checkCIF report

## Figures and Tables

**Table 1:** Hydrogen-bond geometry (Å, °)

*D*—H⋯*A*	*D*—H	H⋯*A*	*D*⋯*A*	*D*—H⋯*A*
N—H0*A*⋯O1^i^	0.86	2.04	2.864 (5)	159
C1—H1*A*⋯O3	0.96	2.16	2.882 (5)	131
C6—H6*A*⋯O1^i^	0.96	2.58	3.401 (6)	143
C7—H7*A*⋯O2^ii^	0.93	2.60	3.525 (6)	176

Symmetry codes: (i) 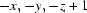; (ii) 
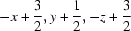
.

**Fig. 1.**
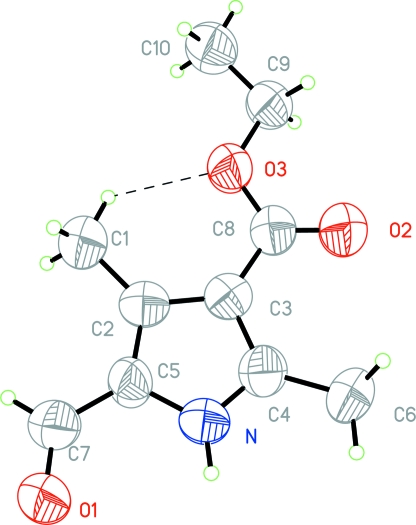
The molecular structure of (I), with displacement ellipsoids for the non-H atoms drawn at the 30% probability level. The short intramolecular C—H···O interaction is shown as dashed line.

## supplementary crystallographic information

### Comment

As part of our owning studies of pyrrole derivatives (Sun *et al.*, 2002), we report here the crystal structure of the title compound, (I), (Fig. 1), which is approximately planar (for the non-hydrogen atoms, r.m.s. deviation from the mean plane = 0.038Å).

A network of N—H···O and C—H···O hydrogen bonds (Table 1) helps to establish the crystal packing in (I). A short intramolecular C—H···O contact also occurs, based on the geometrically positioned H1A atom, which lies on the mirror plane.

### Experimental

A mixture of 2-*tert*-butyl 4-ethyl 3,5-dimethyl-1*H*-pyrrole-2,4-dicarboxylate (30 mmol) in trifluoroacetic acid (40 ml) was stirred for 5 minutes and warmed to 313 K. The mixture was then cooled to 268 K and triethyl orthoformate (45 mmol) was added all at once. The mixture was stirred for about 1 minute, removed from the cold bath and then stirred for 1 h. The trifluoroacetic acid was removed by rotary evaporation and the residue was put into 200 g of ice. The gray floating precipitate was collected by vacuum filtration and washed with 40 ml water then recrystallized twice from ethyl acetate containing Darco carbon black to give 3.7 g of the title compound (Tang *et al.*,1999). Colourless needles of (I) were obtained by slow evaporation of an ethanol solution.

### Refinement

The H atoms were positioned geometrically (N—H = 0.86 Å, C—H = 0.93 and 0.96 Å) and refined as riding with U_iso_(H) =1.2U_eq_(carrier) or 1.5U_eq_(methyl C).

### Crystal data

**Table:**

C_10_H_13_NO_3_	*F*_000_ = 416
*M**_r_* = 195.21	*D*_x_ = 1.299 Mg m^−^^3^
Monoclinic, *P*2_1_/*n*	Mo *K*α radiation λ = 0.71073 Å
Hall symbol: -P 2yn	Cell parameters from 25 reflections
*a* = 3.9830 (8) Å	θ = 9–12º
*b* = 15.572 (3) Å	µ = 0.10 mm^−^^1^
*c* = 16.213 (3) Å	*T* = 293 (2) K
β = 96.96 (3)º	Needle, colourless
*V* = 998.2 (3) Å^3^	0.20 × 0.05 × 0.05 mm
*Z* = 4	

### Data collection

**Table:**

Enraf–Nonius CAD-4 diffractometer	*R*_int_ = 0.021
Radiation source: fine-focus sealed tube	θ_max_ = 25.2º
Monochromator: graphite	θ_min_ = 1.8º
*T* = 293(2) K	*h* = −4→4
ω/2θ scans	*k* = 0→18
Absorption correction: ψ scan(North *et al.*, 1968)	*l* = 0→19
*T*_min_ = 0.981, *T*_max_ = 0.995	3 standard reflections
2069 measured reflections	every 200 reflections
1798 independent reflections	intensity decay: none
935 reflections with *I* > 2σ(*I*)	

### Refinement

**Table:**

Refinement on *F*^2^	Secondary atom site location: difference Fourier map
Least-squares matrix: full	Hydrogen site location: inferred from neighbouring sites
*R*[*F*^2^ > 2σ(*F*^2^)] = 0.083	H-atom parameters constrained
*wR*(*F*^2^) = 0.190	*w* = 1/[σ^2^(*F*_o_^2^) + (0.06*P*)^2^ + 0.5*P*] where *P* = (*F*_o_^2^ + 2*F*_c_^2^)/3
*S* = 1.03	(Δ/σ)_max_ < 0.001
1798 reflections	Δρ_max_ = 0.16 e Å^−^^3^
127 parameters	Δρ_min_ = −0.17 e Å^−^^3^
Primary atom site location: structure-invariant direct methods	Extinction correction: none

### Special details

**Table:**

Geometry. All e.s.d.'s (except the e.s.d. in the dihedral angle between two l.s. planes) are estimated using the full covariance matrix. The cell e.s.d.'s are taken into account individually in the estimation of e.s.d.'s in distances, angles and torsion angles; correlations between e.s.d.'s in cell parameters are only used when they are defined by crystal symmetry. An approximate (isotropic) treatment of cell e.s.d.'s is used for estimating e.s.d.'s involving l.s. planes.
Refinement. Refinement of *F*^2^ against ALL reflections. The weighted *R*-factor *wR* and goodness of fit *S* are based on *F*^2^, conventional *R*-factors *R* are based on *F*, with *F* set to zero for negative *F*^2^. The threshold expression of *F*^2^ > σ(*F*^2^) is used only for calculating *R*-factors(gt) *etc*. and is not relevant to the choice of reflections for refinement. *R*-factors based on *F*^2^ are statistically about twice as large as those based on *F*, and *R*- factors based on ALL data will be even larger.

### Fractional atomic coordinates and isotropic or equivalent isotropic displacement parameters (Å^2^)

**Table:**

	*x*	*y*	*z*	*U*_iso_*/*U*_eq_	
N	0.3042 (9)	−0.0589 (2)	0.60096 (19)	0.0712 (11)	
H0A	0.1844	−0.0604	0.5531	0.085*	
O1	0.0871 (10)	0.11115 (17)	0.55062 (19)	0.0982 (12)	
C1	0.7132 (14)	0.0521 (3)	0.7869 (3)	0.0900 (16)	
H1A	0.8505	0.0210	0.8296	0.135*	
H1B	0.5247	0.0773	0.8096	0.135*	
H1C	0.8459	0.0965	0.7656	0.135*	
O2	0.8180 (10)	−0.23224 (19)	0.76904 (18)	0.0989 (12)	
C2	0.5870 (11)	−0.0078 (3)	0.7184 (2)	0.0619 (11)	
O3	0.9364 (8)	−0.11553 (16)	0.84573 (16)	0.0802 (10)	
C3	0.6177 (10)	−0.0982 (2)	0.7165 (2)	0.0575 (10)	
C4	0.4352 (13)	−0.1260 (3)	0.6409 (3)	0.0781 (14)	
C5	0.3840 (12)	0.0144 (2)	0.6459 (2)	0.0719 (13)	
C6	0.3837 (13)	−0.2149 (3)	0.6079 (3)	0.0840 (15)	
H6A	0.2464	−0.2132	0.5550	0.126*	
H6B	0.2725	−0.2487	0.6460	0.126*	
H6C	0.5989	−0.2401	0.6015	0.126*	
C7	0.2893 (14)	0.0965 (3)	0.6142 (3)	0.0825 (15)	
H7A	0.3857	0.1437	0.6432	0.099*	
C8	0.7874 (12)	−0.1552 (3)	0.7771 (2)	0.0656 (11)	
C9	1.1202 (13)	−0.1682 (3)	0.9080 (2)	0.0797 (14)	
H9A	1.3017	−0.1981	0.8852	0.096*	
H9B	0.9713	−0.2105	0.9283	0.096*	
C10	1.2602 (13)	−0.1105 (3)	0.9765 (3)	0.0888 (15)	
H10A	1.3865	−0.1437	1.0195	0.133*	
H10B	1.0782	−0.0815	0.9988	0.133*	
H10C	1.4063	−0.0688	0.9556	0.133*	

### Atomic displacement parameters (Å^2^)

**Table:**

	*U*^11^	*U*^22^	*U*^33^	*U*^12^	*U*^13^	*U*^23^
N	0.073 (3)	0.071 (2)	0.0652 (18)	0.004 (2)	−0.0076 (18)	0.0032 (16)
O1	0.125 (3)	0.0727 (18)	0.0864 (19)	0.006 (2)	−0.029 (2)	0.0039 (15)
C1	0.110 (5)	0.072 (3)	0.086 (3)	−0.002 (3)	0.001 (3)	−0.004 (2)
O2	0.130 (4)	0.0705 (19)	0.092 (2)	0.009 (2)	−0.004 (2)	−0.0027 (15)
C2	0.053 (3)	0.076 (3)	0.0595 (19)	0.001 (2)	0.0175 (18)	0.0012 (18)
O3	0.094 (3)	0.0629 (16)	0.0791 (18)	0.0052 (19)	−0.0082 (17)	0.0004 (14)
C3	0.047 (3)	0.069 (2)	0.060 (2)	−0.007 (2)	0.0210 (18)	0.0007 (17)
C4	0.088 (4)	0.069 (3)	0.077 (3)	−0.020 (3)	0.007 (2)	0.002 (2)
C5	0.071 (3)	0.055 (2)	0.083 (3)	0.010 (2)	−0.017 (2)	−0.002 (2)
C6	0.094 (4)	0.071 (3)	0.086 (3)	−0.011 (3)	0.003 (3)	−0.017 (2)
C7	0.101 (4)	0.075 (3)	0.069 (2)	−0.009 (3)	0.001 (3)	0.003 (2)
C8	0.061 (3)	0.065 (2)	0.074 (2)	0.002 (3)	0.020 (2)	−0.002 (2)
C9	0.090 (4)	0.068 (2)	0.077 (3)	0.012 (3)	−0.003 (3)	0.003 (2)
C10	0.084 (4)	0.089 (3)	0.091 (3)	0.012 (3)	−0.003 (3)	0.007 (2)

### Geometric parameters (Å, °)

**Table:**

N—C4	1.304 (5)	C3—C8	1.432 (5)
N—C5	1.370 (5)	C4—C6	1.489 (5)
N—H0A	0.8600	C5—C7	1.412 (5)
O1—C7	1.250 (5)	C6—H6A	0.9600
C1—C2	1.490 (5)	C6—H6B	0.9600
C1—H1A	0.9600	C6—H6C	0.9600
C1—H1B	0.9600	C7—H7A	0.9300
C1—H1C	0.9600	C9—C10	1.484 (5)
O2—C8	1.214 (4)	C9—H9A	0.9700
C2—C5	1.388 (5)	C9—H9B	0.9700
C2—C3	1.414 (5)	C10—H10A	0.9600
O3—C8	1.346 (4)	C10—H10B	0.9600
O3—C9	1.431 (4)	C10—H10C	0.9600
C3—C4	1.415 (5)		
			
C4—N—C5	110.5 (3)	C4—C6—H6B	109.5
C4—N—H0A	124.7	H6A—C6—H6B	109.5
C5—N—H0A	124.7	C4—C6—H6C	109.5
C2—C1—H1A	109.5	H6A—C6—H6C	109.5
C2—C1—H1B	109.5	H6B—C6—H6C	109.5
H1A—C1—H1B	109.5	O1—C7—C5	125.6 (4)
C2—C1—H1C	109.5	O1—C7—H7A	117.2
H1A—C1—H1C	109.5	C5—C7—H7A	117.2
H1B—C1—H1C	109.5	O2—C8—O3	120.2 (4)
C5—C2—C3	105.8 (3)	O2—C8—C3	125.7 (4)
C5—C2—C1	125.8 (4)	O3—C8—C3	114.0 (3)
C3—C2—C1	128.1 (4)	O3—C9—C10	107.2 (3)
C8—O3—C9	117.2 (3)	O3—C9—H9A	110.3
C2—C3—C4	106.7 (3)	C10—C9—H9A	110.3
C2—C3—C8	129.5 (4)	O3—C9—H9B	110.3
C4—C3—C8	123.7 (4)	C10—C9—H9B	110.3
N—C4—C3	108.5 (3)	H9A—C9—H9B	108.5
N—C4—C6	122.5 (4)	C9—C10—H10A	109.5
C3—C4—C6	129.0 (4)	C9—C10—H10B	109.5
N—C5—C2	108.5 (3)	H10A—C10—H10B	109.5
N—C5—C7	121.8 (3)	C9—C10—H10C	109.5
C2—C5—C7	129.5 (4)	H10A—C10—H10C	109.5
C4—C6—H6A	109.5	H10B—C10—H10C	109.5
			
C5—C2—C3—C4	1.3 (5)	C1—C2—C5—N	−176.2 (4)
C1—C2—C3—C4	175.6 (5)	C3—C2—C5—C7	−175.8 (5)
C5—C2—C3—C8	−177.2 (4)	C1—C2—C5—C7	9.7 (8)
C1—C2—C3—C8	−2.9 (8)	N—C5—C7—O1	11.4 (8)
C5—N—C4—C3	−0.7 (5)	C2—C5—C7—O1	−175.2 (5)
C5—N—C4—C6	178.6 (5)	C9—O3—C8—O2	−1.6 (7)
C2—C3—C4—N	−0.4 (5)	C9—O3—C8—C3	−178.1 (4)
C8—C3—C4—N	178.2 (4)	C2—C3—C8—O2	−175.8 (5)
C2—C3—C4—C6	−179.6 (5)	C4—C3—C8—O2	5.8 (8)
C8—C3—C4—C6	−0.9 (8)	C2—C3—C8—O3	0.4 (7)
C4—N—C5—C2	1.6 (5)	C4—C3—C8—O3	−177.9 (4)
C4—N—C5—C7	176.2 (5)	C8—O3—C9—C10	179.8 (4)
C3—C2—C5—N	−1.7 (5)		

### Hydrogen-bond geometry (Å, °)

**Table:**

*D*—H···*A*	*D*—H	H···*A*	*D*···*A*	*D*—H···*A*
N—H0A···O1^i^	0.86	2.04	2.864 (5)	159
C1—H1A···O3	0.96	2.16	2.882 (5)	131
C6—H6A···O1^i^	0.96	2.58	3.401 (6)	143
C7—H7A···O2^ii^	0.93	2.60	3.525 (6)	176

Symmetry codes: (i) −*x*, −*y*, −*z*+1; (ii) −*x*+3/2, *y*+1/2, −*z*+3/2.
